# Investigation of the anti-hyperglycemic and antioxidant effects of wheat bread supplemented with onion peel extract and onion powder in diabetic rats

**DOI:** 10.1007/s40200-021-00770-x

**Published:** 2021-03-08

**Authors:** Sara Masood, Attiq ur Rehman, Shahid Bashir, Mohamed El Shazly, Muhammad Imran, Palwasha Khalil, Faiza Ifthikar, Hafiza Madiha Jaffar, Tara Khursheed

**Affiliations:** 1 University Institute of Diet and Nutritional Sciences (UIDNS), Faculty of Allied Health Sciences, University of Lahore, Lahore, Pakistan; 2 Horticulture Technologies, Production Systems Unit, Natural Resources Institute (Luke), Toivonlinnantie 518, FI-21500 Piikkiö, Finland; 3 Department of Agricultural Sciences, Faculty of Agriculture and Forestry, University of Helsinki, FI-00790 Helsinki, Finland; 4 Department of Pharmaceutical Biology, Faculty of Pharmacy and Biotechnology, German University in Cairo, Cairo, Egypt; 5 Department of Pharmacognosy, Faculty of Pharmacy, Ain Shams University, African Union Organization Street, Cairo, Abbassia 11566 Egypt

**Keywords:** Onion powder, Onion peel extract, Antioxidant activity, Hypoglycemic activity, Wheat bread, Phytochemicals

## Abstract

**Aim:**

Onion is one of the commonly cultivated and consumed vegetables rich in nutrients and phytochemicals. Various nutraceuticals are found in the outer fleshy layers and dry peel of onion which usually is treated as a common biowaste. Diabetes mellitus is a leading non communicable disease causing hyperglycemia and increased production of free radicals that potentially disrupts antioxidant enzymatic activity. Considering global consumption of wheat, the present study was designed to evaluate the anti-hyperglycemic and antioxidant effects of wheat bread supplemented with onion peel extract (OPE) or onion powder (OP) on diabetic rats.

**Methods:**

In this study, ethanolic extract of onion peel and onion bulb were prepared separately. Male Sprague Dawley rats were divided into 6 groups (*n* = 7). Different regimens of supplemented wheat bread (OPE (1% and 3%) and OP (5% and 7%)) were given to diabetic rats for eight weeks, plain bread was used as the control. Blood glucose level, body weight and activities of SOD, CAT, GPx, GR, GSH and MDA in the liver and kidney tissues were evaluated. Statistical analysis was performed using SPSS Version (25) and Dunnett’s multiple comparison test.

**Results:**

Bread supplemented with 1% and 3% onion peel extract and 7% onion powder significantly reduced blood glucose levels and MDA in the treated rats compared with the control group diabetic rats. Body weight of diabetic rats was reduced for control group, while onion supplemented diet improved the body weight of treated rats. Onion supplementation also brought significant improvement in antioxidant enzyme activities among the treated diabetic rats.

**Conclusion:**

These findings suggested that onion supplementation is effective in lowering blood glucose and could potentially aid in protecting organs from oxidative stress.

## Introduction

Diabetes is characterized by impaired carbohydrate, protein, and fat metabolism that results from poor insulin secretion, insulin resistance, or both. Diabetes mellitus is currently one of the most prevalent and life-threatening metabolic disorder globally. Based on several estimations, it is suggested that its incidence rate will rise even more rapidly in the coming future [1]. Recurrent hyperglycemia, which is the major clinical manifestation in diabetic patients, is primarily associated with the progression of several acute and chronic diabetic complications. Keeping blood glucose under controlled levels, understanding the regulation of insulin response, and preventing diabetes-associated complications are the most important goals in managing diabetes [2]. Oxidative stress is one of the primary factors involved in the development of diabetes complications and is postulated to be linked with increased production of oxygen free radical [3]. Under normal physiological conditions, various antioxidant defense mechanisms work to protect the body from the dangers of free radical production [4]. In diabetes, the elevation of blood glucose levels promotes the production of reactive oxygen species (ROS) [5]. The mechanisms involved in ROS production not only includes auto-oxidative and non-enzymatic glycosylation but also the metabolic stress that results from altered energy metabolism, raised levels of inflammatory mediators, and poor status of the antioxidant defense system [6].

To understand the mechanism of diabetes and its relation to oxidative stress, several in vivo models were developed including alloxan-induced diabetes in mice. Alloxan, a pyrimidine derivative, is used to induce diabetes and produce cytotoxic free radicals that destroy animals’ pancreatic cells. These effects suggest that alloxan could influence the intrinsic antioxidant defense system in response to oxidative stress [7]. The hazardous effects of oxygen and hydroxy radicals can be prevented by various antioxidant enzymes such as catalase (CAT), glutathione peroxidase (GPx), and superoxide dismutase (SOD). Glutathione (GSH) also works efficiently to neutralize the damaging electrophiles [8].

Several classes of drugs were developed to manage diabetes showing impressive results but many of the developed products resulted in side effects because the antidiabetic drugs are lifelong. Therefore, there is growing interest to incorporate medicinal plant-based products into the diet of the general population to reduce the medication use and thus their side effects and to obtain maximum preventive outcome [9]. Onion (*Allium cepa* L.) is a widely cultivated and consumed vegetable that serves as an excellent source of macro and micronutrients [10]. Along with the bulb, the peel of onion, which is generally regarded as a biowaste is rich in various phytonutrients. Although the management of diabetes with traditional plants has been a widespread practice for centuries [11], only a few remedies have been assessed scientifically. Many researchers demonstrated the antidiabetic, hypocholesterolemic, and antioxidant potential of onion peel extract and onion powder [12–18]. The evaluation and utilization of biowaste (onion peel) as a supplementary ingredient in food for target groups will have sustainable health, economic and environmental impact. Wheat (*Triticum aestivum* L.) is one of the most commonly consumed staple food [19], and there has been growing interest towards its fortification with micronutrients and nutraceuticals [20–22]. Hence, in the present study, the American Institute of Nutrition (AIN)-93G diet for rodents was modified to formulate bread using ingredients like wheat flour, onion powder/onion peel extract to evaluate the effect of their supplementation on blood glucose level, and the antioxidant activities of various enzymes in the kidney and liver homogenates of diabetic rats. The potential physiological influence of the onion’s outer peel and the edible bulb has not been compared yet in a scientific study. Considering all these points, the present study examined the potential impact and antioxidant activities of the peel extract and bulb powder of onion on the in vivo kidney and liver cell metabolism of diabetic rats.

## Material and Methods

### Animals

Male Sprague Dawley rats (10–12 weeks old) weighing 250–300 g, were purchased from the National Institute of Health (NIH) Islamabad-Pakistan and were acclimatized for 2 weeks in the animal house of the University of Lahore, Pakistan (March 2019–May 2019). Seven rats were kept in each cage and the environmental conditions such as humidity (55 ± 5%) and temperature (23 ± 2 °C) were sustained during the entire study with 12 h of light-dark period. Rats were given free access to the basal diet and water ad libitum. The rats were handled according to the international guidelines on the use and handling of experimental animals (US National Institute of Health) [23] and the guidelines provided by the ethical committee of the University of Lahore, Pakistan (Ref No: IRB-UOL-FAHS/435/2019).

### Onion peel extract preparation

Excellent quality, even-sized, undamaged onions (Var. Desi Red, Phulkara) were procured from the local market. The outer dry and fleshy layers of onions were washed thoroughly using sterile water. Extraction was done with 60% ethanol at 50 °C for 3 h [13]. The extract was filtered, concentrated, and stored after freeze-drying. The extraction yield was 5.35 ± 0.12% while the total phenolic content was 397.85 ± 4.03 mg/g as determined by the Folin-Ciocalteu method [24].

### Onion powder preparation

The obtained peeled onion bulbs were chopped using a sterile knife and placed in a drying oven at 50 °C for 3 days. The dried onion pieces were grounded into a fine powder using an electric grinder and the obtained powder was stored in an airtight container inside the refrigerator [25]. The yield of onion powder was 12 g/100 g of the raw onion.

### Preparation of bread

Five different formulations of bread were prepared including (1) a control bread (B_0_) based on the basic AIN-93G diet without any supplementation; (2) bread with 1% onion peel extract (B_1_); (3) bread with 3% onion peel extract (B_2_); (4) bread with 5% onion powder (B_3_); and (5) bread with 7% onion powder (B_4_). The preparation of all mentioned types of bread was done by following the same procedure [26] and the composition of each bread is presented in Table 1.

**Table 1:** Ingredients (g/100 g) of the experimental diets (breads) fed to rats for 56 days

Composition	Control Bread (B_0_)	Bread with 1% OPE (B_1_)	Bread with 3% OPE (B_2_)	Bread with 5% onion powder (B_3_)	Bread with 7% onion powder (B_4_)
Wheat	68.2	67.2	65.2	63.2	61.2
Salt	1.36	1.36	1.36	1.36	1.36
Dry yeast	1.36	1.36	1.36	1.36	1.36
Improver	0.68	0.68	0.68	0.68	0.68
Casein	14.58	14.58	14.58	14.58	14.58
Soybean oil	8.93	8.93	8.93	8.93	8.93
Minerals	3.38	3.38	3.38	3.38	3.38
Vitamin mix	0.97	0.97	0.97	0.97	0.97
L-cysteine	0.29	0.29	0.29	0.29	0.29
Choline	0.24	0.24	0.24	0.24	0.24
TBHQ	0.001	0.001	0.001	0.001	0.001
Onion powder	0	0	0	5	7
Onion peel extract	0	1	3	0	0

The control wheat bread comprised 100% wheat flour, 70% water (vol/wt, flour basis), 2% dry yeast (wt/wt, flour basis) (Rossmoor, Pakistan), 2% salt (wt/wt, flour basis), and 1% dough improver (wt/wt, flour basis) (eka-300) [26]. For supplemented bread, the formulation was the same but for bread B_1_ and B_2,_ the wheat flour was substituted with OPE at 1% and 3%, respectively. Whereas for bread B_3_ and B_4_ the wheat flour was substituted with onion powder at 5% and 7%, respectively [13, 27–29].

### Induction of diabetes in rats

Diabetes was induced in overnight fasted Sprague Dawley rats by injecting alloxan (120 mg/kg/BW) dissolved in normal saline, through the subcutaneous route. Glucose solution (20% (*w*/*v*)) was also given to these rats to prevent fatal post-alloxan hypoglycemia. After 48 h of alloxan administration, the rats with fasting blood glucose levels>250 mg/dl were considered diabetic and were included in the study [18, 30].

#### Treatment groups

The animals were randomly divided into six groups (7 rats each):

► Group 1 (**NC**): Normal rats fed with control bread.

► Group 2 (**DC**): Diabetic rats fed with control bread.

► Group 3 (**OPE_1**): Diabetic rats fed with bread supplemented with OPE (1%).

► Group 4 (**OPE_3**): Diabetic rats fed with bread supplemented with OPE (3%).

► Group 5 (**OP_5**): Diabetic rats fed with bread supplemented with onion powder (5%).

► Group 6 (**OP_7**): Diabetic rats fed with bread supplemented with onion powder (7%).

Before starting the trial, fasting blood glucose level (mg/dl) and body weight (g) were determined using a glucometer [31] and weighing scale, respectively. The modified diet was then given to the rats for 8 weeks. During the study, physical parameters such as water and bread consumption were measured daily (Appendix Table 4) while blood glucose was determined fortnightly. At the end of the trial, the rats fasted for 12 h and the final measurement of blood glucose level and body weight was taken [32]. All rats were anesthetized and sacrificed as per ethical committee guidelines and at 4 °C, the kidney and liver tissues were excised. Ice-cold saline was used to wash the isolated tissues that were later dipped in liquid nitrogen and instantly stored at −80 °C until further analysis [33].

### Biochemical analysis

In the liver and kidney tissue homogenates, the activity of superoxide dismutase (SOD) was determined by the Misra and Fridovich method using a Hitachi U-2000 spectrophotometer for 4 min at 480 nm. The potential enzymatic activity is stated based on its ability to inhibit the epinephrine oxidation up to 50%, that is equivalent to 1 U per milligram of protein [34, 35]. The method of Aebi was used to assess the activity of catalase (CAT) in which absorbance was determined by UV spectrophotometer at 240 nm for 1 min [35, 36]. The method elaborated by Carlberg and Mannervik was used to determine the activity of Glutathione reductase (GR) and recommendations of Akerboom and Sies were followed to measure the reduced glutathione (GSH) concentration in the kidney and liver homogenates [37–40]. Hepatic and renal enzymatic activity of glutathione peroxidase (GPx) was evaluated following the Flohe and Gunzler method, using cumene hydrogen peroxide while the absorbance was calibrated at 340 nm [41, 42]. The procedure described by Ohkawa, Ohishi, and Yagi was used to determine the concentration of malondialdehyde (MDA), which indicates the extent of lipid peroxidation [43, 44]. Enzymatic activities were reported as per milligram of protein and the protocol of Lowry, Rosebrough, Farr, and Randall was used for estimation of tissue protein content using bovine serum albumin (BSA) as the standard [45, 46].

### Statistical analysis

Data were analyzed using SPSS (Version 25.0) [47]. Dunnett’s multiple comparison test (*p* < 0.01) was performed to compare the antioxidants and enzymatic activity between the diabetic and control (DC) groups [33, 48].

## Results

### Effect of OPE and OP supplemented bread on the bodyweight of diabetic rats

The results obtained in the current study indicated that the rat’s body weight (BW) was substantially reduced after induction of diabetes. The incorporation of bread supplemented with onion peel or onion powder significantly improved the body weight, although the effect was statistically significant by onion peel supplementation (Table 2). The difference between the mean body weight of the treated and diabetic control group was initially insignificant (day 0) but during the later stages of the trial (day 28 and 56), a statistically significant increase in body weight of the treatment groups was observed (*p* < 0.01) (Table 2).

**Table 2:** Effects of onion peel extract and onion powder supplemented breads on body weight changes in diabetic rats

Groups	Body Weight (g)	Day 28	Day 56
	Day 0		
NC	257 ± 2.5	276 ± 3.0*	295 ± 1.4*
DC	258 ± 3.2	237 ± 2.1	222 ± 1.7
OPE_1	266 ± 4.2	267 ± 4.6*	270 ± 4.2*
OPE_3	266 ± 6.7	271 ± 6.2*	282 ± 6.1*
OP_5	262 ± 6.2	256 ± 6.0*	259 ± 5.9*
OP_7	258 ± 5.6	262 ± 5.7*	266 ± 6.2*

Values expressed as means ± SEM, (NC = normal control, DC = diabetic control, OPE_1 = group fed with bread containing 1% onion peel extract, OPE_3 = group fed with bread containing 3% onion peel extract, OP_5 = group fed with bread containing 1% onion powder, OP_7 = group fed with bread containing 7% onion powder), * shows the significant difference compared with the diabetic control group (*p* < 0.01) via Dunnett’s multiple comparison test

### Effect of OPE and OP supplemented bread on blood glucose level of diabetic rats

Blood glucose levels were measured fortnightly and weekly variation in all the groups is presented in Table 3. The results showed a significant effect of the bread incorporated with onion peel extract and onion powder on the blood glucose level of alloxan-induced diabetic rats. The initial and final blood glucose levels in diabetic rats that were fed with the control bread did not vary significantly. In treatment groups that were either fed with 1%, OPE supplemented bread, 3% OPE supplemented bread, or 7% OP supplemented bread, the final blood glucose level (day 56) was significantly lower (*p* < 0.01) than the initial blood glucose levels (day 0). While comparing the treatment groups, it can be seen that in OPE_3 group blood glucose level dropped remarkably from 289 ± 5.8 on day 0 to 205 ± 3.3 on day 56 which is significantly high as compared to OPE_1 and OP_7 groups where the final day blood glucose level was 234 ± 6.2 and 230 ± 3.3 respectively. Whereas the glucose-lowering effect of 5% OP supplemented bread remained non-significant for most of the observation checkpoints except for the end-point measurement (day 56) (*p* < 0.01). The overall glucose-lowering effect of 3% OPE supplemented bread was more pronounced as compared to any other supplemented bread.

**Table 3:** Effects of onion peel extract and onion powder supplemented breads on blood glucose level in diabetic rats

Groups	Blood glucose level (mg/dl)				
	Day 0	Day 14	Day 28	Day 42	Day 56
NC	82 ± 0.4*	86 ± 0.9*	87 ± 1.0*	93 ± 0.6*	93 ± 0.7*
DC	285 ± 5.8	288 ± 6.3	289 ± 5.7	290 ± 6.1	294 ± 4.8
OPE_1	285 ± 7.9	280 ± 7.8	257 ± 5.9*	241 ± 3.3*	234 ± 6.2*
OPE_3	289 ± 5.8	275 ± 4.6	242 ± 4.4*	219 ± 3.4*	205 ± 3.3*
OP_5	289 ± 2.6	284 ± 2.7	280 ± 2.8	277 ± 2.8	268 ± 3.5*
OP_7	292 ± 5.4	282 ± 5.0	264 ± 5.2*	246 ± 4.5*	230 ± 3.3*

Values expressed as means ± SEM, (NC = normal control, DC = diabetic control, OPE_1 = group fed with bread containing 1% onion peel extract, OPE_3 = group fed with bread containing 3% onion peel extract, OP_5 = group fed with bread containing 1% onion powder, OP_7 = group fed with bread containing 7% onion powder), * shows the significant difference compared to the diabetic control group (*p* < 0.01) via Dunnett’s multiple comparison test

### Effect of OPE and OP supplemented bread on the antioxidant enzyme activities and MDA levels of diabetic rats

**Fig. 1:**
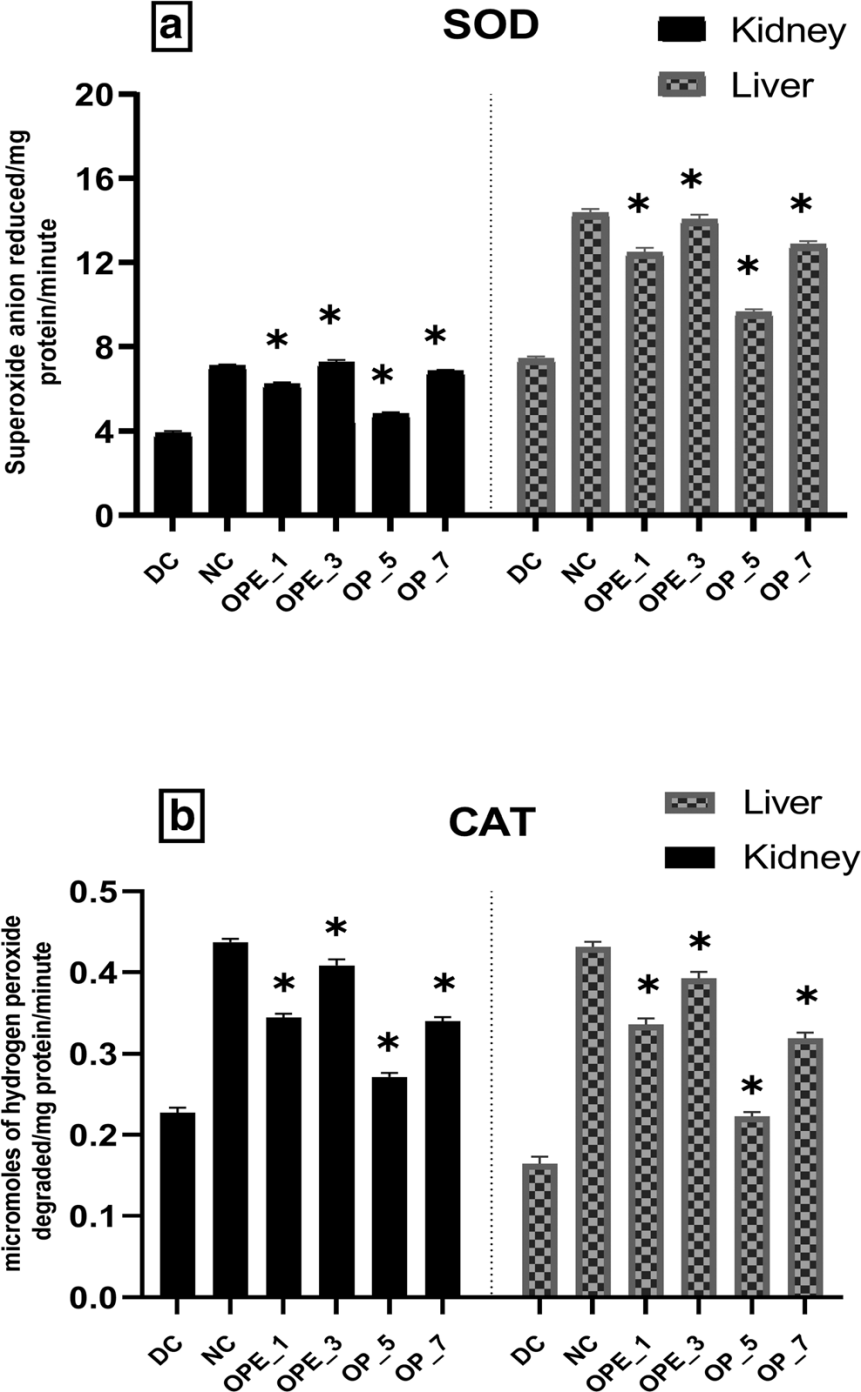
**Effects of onion peel extract and onion powder supplemented bread on SOD (a) and CAT (b) activities in the liver and kidney tissues of normal and experimental rats. **NC = normal control, DC = diabetic control, OPE_1 = group fed with bread containing 1% onion peel extract, OPE_3 = group fed with bread containing 3% onion peel extract, OP_5 = group fed with bread containing 1% onion powder, OP_7 = group fed with bread containing 7% onion powder. *shows the significant difference compared with the diabetic control group (*p* < 0.01) via Dunnett’s multiple comparison test, while ‘ns’ shows a non-significant difference

**Fig. 2:**
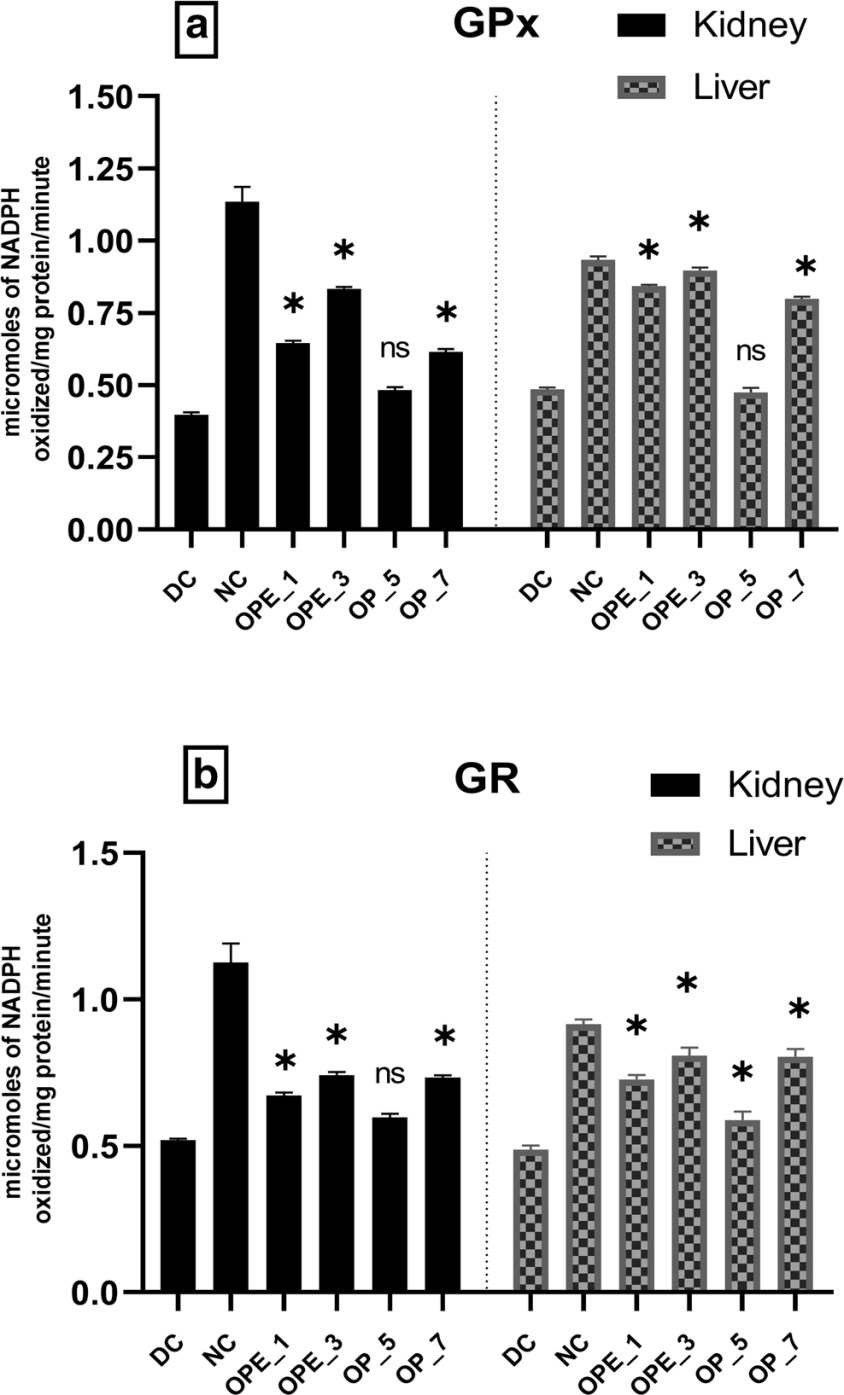
**Effects of onion peel extract and onion powder supplemented bread on GPx (a) and GR (b) activities in the liver and kidney tissues of normal and experimental rats. **NC = normal control, DC = diabetic control, OPE_1 = group fed with bread containing 1% onion peel extract, OPE_3 = group fed with bread containing 3% onion peel extract, OP_5 = group fed with bread containing 1% onion powder, OP_7 = group fed with bread containing 7% onion powder.*shows the significant difference compared with the diabetic control group (*p* < 0.01) via Dunnett’s multiple comparison test, while ‘ns’ shows a non-significant difference

**Fig. 3:**
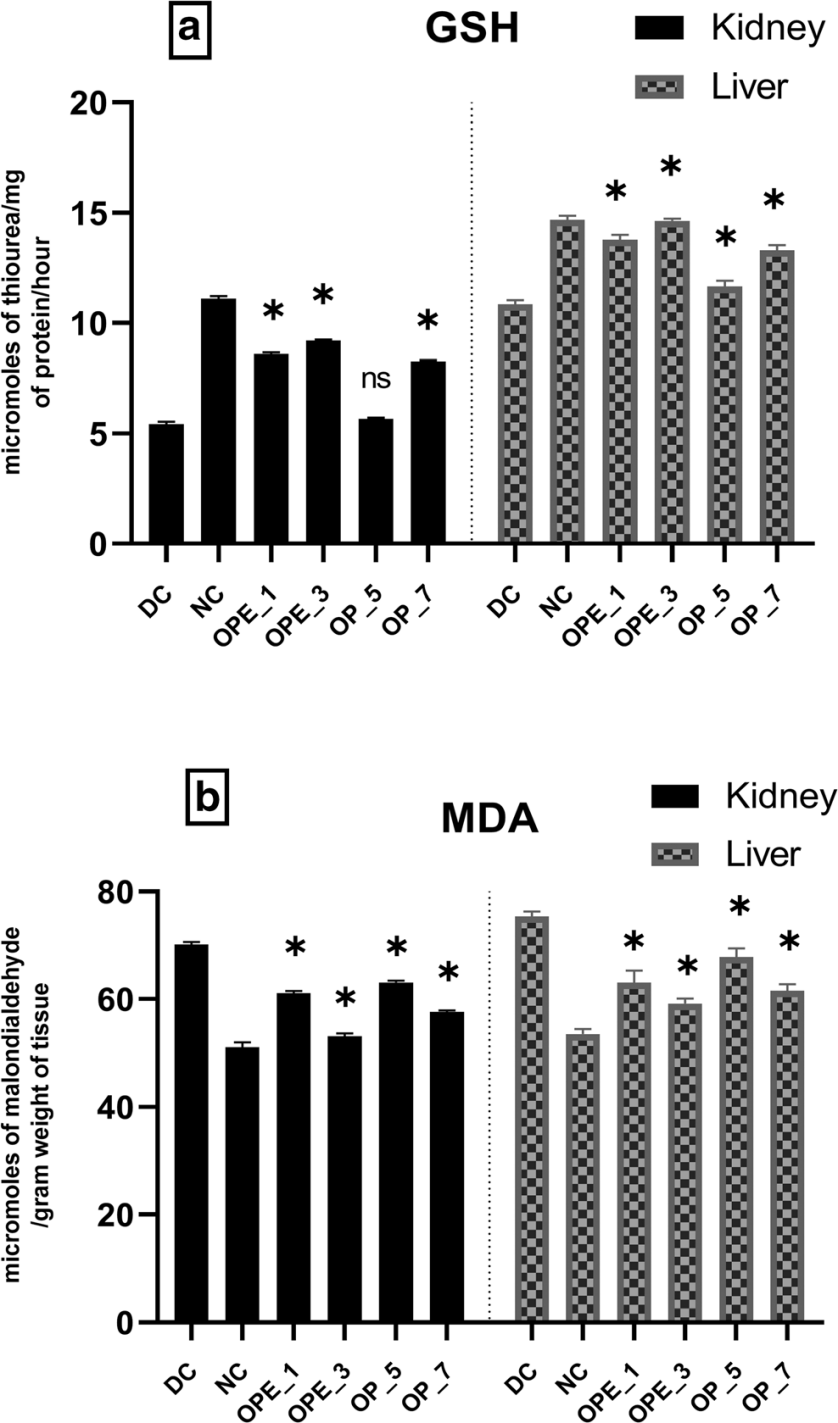
**Effects of onion peel extract and onion powder supplemented bread on GSH (a) and MDA (b) levels activities in the liver and kidney tissues of normal and experimental rats. **NC = normal control, DC = diabetic control, OPE_1 = group fed with bread containing 1% onion peel extract, OPE_3 = group fed with bread containing 3% onion peel extract, OP_5 = group fed with bread containing 1% onion powder, OP_7 = group fed with bread containing 7% onion powder.*shows the significant difference compared with the diabetic control group (*p* < 0.01) via Dunnett’s multiple comparison test, while ‘ns’ shows a non-significant difference

Significant (*p* < 0.01) rise in the level of malondialdehyde (MDA) and decline in catalase (CAT), superoxide dismutase (SOD), glutathione reductase (GR), glutathione peroxidase (GPx) activities, and glutathione (GSH) content were reported in the diabetic control group in comparison with the normal rats (Figs. 1, 2 and 3). In the 5% OP supplemented group, no significant changes were observed in GPx levels of the liver and kidney in comparison to the diabetic control group. Also, there was no significant variation in the kidney’s GR and GSH levels by the end of the 56 days treatment. However, in a similar group also supplemented with 5% OP there was a significant 29% and 35% reduction (*p* < 0.01) in the liver’s superoxide dismutase and catalase activity, respectively.

Diabetic rat groups that were treated with OPE (1% and 3%) and OP (7%) showed a significant (*p* < 0.01) increase in the activities of GPx, GR, CAT, SOD, and GSH levels but the effect was more pronounced in the group supplemented with 3% OPE bread. It was observed that MDA levels were reduced by (21%) in the liver of the 3% OPE treated group in comparison with the diabetic control. Along with the significant improvement in GPx, GR, and GSH levels, the similar group presented a prominent threefold increase in SOD and CAT levels in the liver as compared with the diabetic control group showing the restoration of the altered antioxidant defense system near to the normal level.

## Discussion

We observed a significant increase in the blood glucose level with alloxan induction. This might be due to the destruction of Langerhans islets β-cells by alloxan which also elevates oxidative stress by compromising the intrinsic antioxidant mechanism and by increasing the production of free radicals [18, 49, 50]. Oxidative damage induced by reactive oxygen species can promote negative consequences including uncontrolled hyperglycemia [51].

The rats’ body weights were reduced after induction of diabetes (Table 2). This kind of weight reduction was observed in alloxan-induced diabetes in rodents [52]. The breakdown of lipids stored in adipose tissues and the disruption of the skeletal muscle proteins might have contributed to this weight loss [53]. When supplemented bread was added to the diet of alloxan-induced diabetic rats, their body weight increased along with their body’s ability to regulate blood glucose levels. However, the effect was more significant in rats that were given bread supplemented with 3% of onion peel extract.

The effect of onion supplementation in reducing blood glucose level was also reported in some recent studies [54–56]. Phytochemicals such as quercetin and allyl-propyl disulfides found in onion peel and bulb might be responsible for the beneficial effect on blood glucose level by up-regulating the expression of insulin receptors and glucose transporters, improving insulin sensitivity and promoting glucose metabolism in peripheral tissues in diabetic rats [13]. Kim et al. (2011) had reported that supplementation of onion peel extract brought significant reduction in blood glucose level by slowing down the glucose absorption rate via inhibition of sucrase enzyme in the intestinal tract [57]. The uptake of glucose is carried out by series of events from binding by insulin to the receptors present on cell surface, and later the ability of insulin to increase glucose transport inside muscle tissue by GLUT [58]. Hence, it is anticipated that phytochemicals present in onion might improve insulin sensitivity by amending the insulin receptor and glucose transporters expression and by promoting glucose metabolism in peripheral tissues of diabetic rats [13]. In the present research, both onion peel and onion powder supplemented breads brought reduction in blood glucose level. However, it was observed that effect of even smaller percentages of onion peel extract was more pronounced as compared to the relatively higher percentages of onion bulb powder. Previous studies have demonstrated that in vitro antioxidant potential of onion peel is fairly high as compared to the inner bulb [17, 59]. Phytochemicals such as quercetin, isorhamnetin and kaempferol which possess strong antidiabetic potential are much more abundant in the outer dry scales and fleshy peels than the inner layers [60, 61] and this might be the reason behind more potent antihyperglycemic and antioxidant activity of the onion peel as compared to the bulb [62].

Among human subjects, few studies have been conducted which demonstrated that *Allium cepa* possess strong potential in lowering blood glucose level of normal subjects as well as diabetic patients [63–65], but antidiabetic potential of onion bulb and onion peel extract has not been compared previously. Although, the use of onion powder and the peel as food supplements has been analyzed on basis of sensorial acceptability by trained panel indicating a potential use of such supplemented breads in future [66]. Therefore, current study serves as a steppingstone towards the development of onion powder and peel based functional foods. In the present research, the activities of studied enzymes of the antioxidative defense system such as CAT and SOD were significantly reduced while the level of malondialdehyde (MDA) was increased in alloxan-induced diabetic rats. The manifestation of oxidative stress occurs when ROS production increases abruptly, or the ROS-scavenging capacity of the body gets attenuated [67]. In the alloxan-induced diabetic rats, weak antioxidant enzymatic activities, elevated lipid peroxidation, and lowered non-enzymatic antioxidant levels were reported in previous studies [68–71]. To protect the body against ROS-mediated oxidative damage, antioxidant enzymes are released including superoxide dismutase (SOD), catalase (CAT), glutathione peroxidase (GPx), and glutathione reductase (GR) [72].

In the present research, the studied antioxidative defense system enzymes such as CAT and SOD displayed lower activities in the evaluated kidney and liver tissues (Fig. 1) and these results agreed with earlier published results [73, 74]. Excess blood glucose and recurrent non-enzymatic glycation of proteins promote the generation of O_2_ and H_2_O_2_ and this might be the reason behind the reduced activities of CAT and SOD [75]. The limited activation of antioxidant enzymes due to the presence of H_2_O_2_ and other hydrogen radicals was recently reported [76]. Another reason behind the decreased activity of CAT and SOD might be the reduction of the protein expression levels that is a common phenomenon in hyperglycemia [77].

The activity of another important antioxidant enzyme, glutathione peroxidase (GPx), was also reduced in the hepatic and renal tissues of diabetic rats indicating impaired scavenging activity of lipid hydroperoxides and H_2_O_2_. A similar finding was observed in a previous investigation [78]. When diabetic rats were fed with bread supplemented with onion peel extract or onion powder, the altered activities of the antioxidant enzymes such as SOD, CAT, GPx, and GR were significantly restored (*p* < 0.01) (Figs. 1 and 2). The antioxidant potential of various nutraceuticals such as quercetin, isorhamnetin, kaempferol, and allyl-propyl disulfides might have contributed to this effect. All these constituents previously showed a potent effect in reducing blood glucose level and oxidative stress as well by modulating various antioxidant enzymes in diabetic rats [79, 80].

Also, it was observed in our present study that the enzymatic activity of GR was significantly reduced in diabetic rats due to a reduction in its production [81]. In groups receiving 3% of onion peel extract or 7% onion powder, the GR activity was raised significantly (p < 0.01) as shown in Fig. 2. It was previously reported that the plasma antioxidant capacity was improved in diabetic rats following onion treatment [80]. Glutathione (GSH) also serves as a marker of oxidative damage as it is an important non-enzymatic antioxidant agent involved in preserving the integrity of the cellular systems [82]. The present study indicated that in the renal and hepatic tissues of alloxan-induced diabetic rats, the GSH level decreased (Fig. 3). The downfall in tissue GSH levels increases the progression of cellular damage initiated by free radicals. The increased rate by which GSH is used in diabetic rats highlights its important activity against ROS [83]. When diabetic rats were fed with onion supplemented bread it restored the GSH to almost normal levels (Fig. 3) but, the effect was more pronounced in the 3% OPE group as compared with other groups suggesting that onion peel extract can be beneficial against oxidative stress. Previous studies indicated the potent antioxidant potential of onion towards increasing the GSH level and upholding the antioxidant enzymatic activities within the normal range [84, 85].

Lipid peroxidation is another crucial factor that is strongly associated with the integrity of cellular membranes and it helps in evaluating the composition and structure of the tissue lipids. In diabetes, the raised level of free radicals in certain tissues may lead to a rise in the rate of lipid peroxidation [86]. Hence malondialdehyde (MDA), which is a product of lipid peroxidation was measured in tissues of diabetic rats as an indicator of the raised rate of lipid peroxidation in their kidneys and liver [87]. Nonetheless, the elevated MDA level observed in the tissues of alloxan-induced rats was found to be declined after treatment with onion in the form of both onion peel extract as well as onion bulb powder. Another benefit that is offered by onion supplementation was the prevention of membrane lipid peroxidation. Previous studies proved that onion possesses strong hypolipidemic properties [88–91]. The observed decline in the concentration of MDA and the overall improved antioxidant status in the onion peel extract and onion powder treated diabetic rats highlights their strong potential against oxidative stress and lipid peroxidation.

The current study suggested that both onion peel and bulb may have beneficial effects against diabetes by lowering glucose levels and suppressing oxidative stress when given in a certain amount and this holds the hope of a new generation of functional foods. Onion peel is commonly treated as agro-biowaste but it contains useful phytochemicals that need the attention of the scientific community as well as the food industry. However, the present study has some limitations. The phenolic content and antioxidant capacity vary among different onion varieties [92] and the biochemical properties of different wheat flour types can affect the final product. Therefore, standardization of both the constituents is needed before any further in vivo experimentation is planned to account for such variations. In earlier studies, the concentration of various phenolic acids and flavonoids in onion extracts was determined by HPLC [93, 94]. Phenols possess weak thermal stability and the baking process increases the amount of the free phenolic acids and decreases the concentration of bound phenolic compounds [95, 96] and thus it is recommended to assess the concentration of phytochemicals in the developed bread samples as well. Moreover, the current study also suffered from being an animal study, and the obtained findings cannot be exactly applied in humans in varying states of health and with diverse dietary patterns. Therefore, it is suggested to conduct a human trial to examine the postprandial metabolic and appetitive responses to the developed bread. If similar findings are projected to human trials, it will open fascinating possibilities for formulating frequently consumed foods like bread with health-promoting effects.

## Figures and Tables

**Figure:**
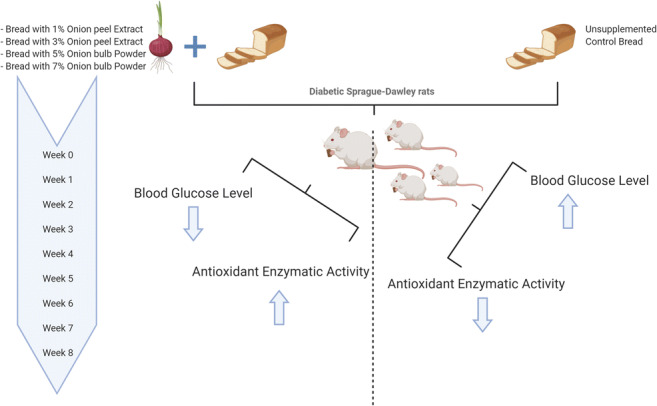
Values presented as the difference between diet provided and the leftover by the end of 24 h for each individual study group; (NC = normal control, DC = diabetic control, OPE_1 = group fed with bread containing 1% onion peel extract, OPE_3 = group fed with bread containing 3% onion peel extract, OP_5 = group fed with bread containing 1% onion powder, OP_7 = group fed with bread containing 7% onion powder).

## Appendix

**Table 4:** Daily feed intake (g) of all the studied rat groups

Days	DC	NC	OPE_1	OPE_1	OP_5	OP_7
Day 1	111	106	110	113	109	110
Day 2	107	105	107	105	104	106
Day 3	112	111	109	111	112	107
Day 4	116	100	101	104	110	105
Day 5	101	105	103	106	107	108
Day 6	109	100	104	111	114	110
Day 7	114	112	109	108	104	111
Day 8	100	105	109	110	107	113
Day 9	115	111	112	111	106	112
Day 10	115	113	114	112	114	110
Day 11	110	110	114	113	117	111
Day 12	113	109	107	110	109	113
Day 13	108	113	115	116	115	116
Day 14	110	108	106	107	111	112
Day 15	111	114	115	116	114	115
Day 16	113	113	116	118	115	113
Day 17	116	114	117	117	114	116
Day 18	112	110	112	113	115	110
Day 19	110	111	117	115	117	113
Day 20	112	110	115	116	112	116
Day 21	116	117	114	115	114	117
Day 22	117	119	117	118	118	118
Day 23	119	118	116	117	117	117
Day 24	111	118	115	118	118	118
Day 25	121	116	117	115	115	115
Day 26	113	114	113	113	113	113
Day 27	110	111	110	116	116	116
Day 28	114	117	116	117	117	115
Day 29	119	121	118	115	116	113
Day 30	118	117	121	119	113	116
Day 31	117	114	114	119	120	116
Day 32	118	115	114	120	117	121
Day 33	117	118	117	118	114	119
Day 34	119	113	115	116	117	115
Day 35	121	118	116	117	114	117
Day 36	115	117	116	120	119	118
Day 37	120	113	114	118	117	121
Day 38	119	115	113	117	113	118
Day 39	120	118	119	114	115	119
Day 40	116	115	116	113	122	118
Day 41	121	119	120	119	116	115
Day 42	122	122	119	118	113	120
Day 43	120	124	121	119	118	119
Day 44	119	116	115	120	118	117
Day 45	122	119	117	122	117	121
Day 46	121	120	121	119	115	118
Day 47	119	121	116	117	115	120
Day 48	122	118	119	118	117	117
Day 49	118	114	115	116	119	117
Day 50	123	121	119	120	118	119
Day 51	126	119	121	118	122	123
Day 52	119	118	122	120	119	118
Day 53	125	119	117	116	116	115
Day 54	118	121	119	118	121	117
Day 55	123	119	121	121	118	121
Day 56	124	118	120	122	117	118
Mean	116.02	114.50	114.74	115.54	114.82	115.40
